# Precision Antifungal Treatment Significantly Extends Voice Prosthesis Lifespan in Patients Following Total Laryngectomy

**DOI:** 10.3389/fmicb.2020.00975

**Published:** 2020-05-20

**Authors:** Daniel R. Pentland, Sarah Stevens, Leila Williams, Mark Baker, Carolyn McCall, Viktorija Makarovaite, Alistair Balfour, Friedrich A. Mühlschlegel, Campbell W. Gourlay

**Affiliations:** ^1^Kent Fungal Group, School of Biosciences, University of Kent, Kent, United Kingdom; ^2^East Kent Hospitals, University NHS Foundation Trust, Kent, United Kingdom; ^3^Laboratoire National de Santé, Dudelange, Luxembourg

**Keywords:** *Candida*, voice prosthesis, laryngectomy, speech therapy, microbiology, antifungal

## Abstract

Indwelling silicone valves called voice prostheses (VPs) are the gold standard for speech rehabilitation in patients with laryngeal cancer following total laryngectomy. Reported VP lifespans amongst these patients are highly variable but when devices fail patients experience loss of voice and an increase risk of chest infection. Early failure of VP is a current clinical concern that is associated with regular hospital visits, reduced quality of life and associated medical cost. Poly-microbial biofilms comprised of both bacterial and fungal microorganisms readily colonize VPs and are linked to loss of device performance and its early failure in addition to providing a reservoir for potential infection. Our detailed analysis of poly-microbial biofilm composition on 159 early failing VPs from 48 total laryngectomy patients confirmed *Candida albicans* as the predominant fungal species and *Staphylococcus aureus* as the most common bacterial colonizer within our patient cohort. Using a combination of microbiological analysis, patient data and a high-throughput antifungal test assay mimicking *in vivo* conditions we established an evidence based precision antifungal treatment approach to VP management. Our approach has allowed us to implement a personalized VP management pathway, which increases device *in situ* lifespan by an average of 270%. Our study represents a significant step forward in both our understanding of the cause of VP failure and a new effective treatment pathway that offers tangible benefit to patients.

## Introduction

Head and neck cancer is the sixth most common cancer worldwide and the eighth most common cancer in the United Kingdom with 650,000 new cases (12,000 in the United Kingdom) and 350,000 deaths (4,000 in the United Kingdom) attributed to it each year worldwide (Leonhard and Schneider-Stickler, 2015; Cancer Research UK, 2018). Laryngeal malignancy is the most frequent head and neck cancer, making up 26.2% of newly diagnosed male cases and 13.1% of female cases in the United Kingdom (Cancer Research UK, 2018). Laryngeal malignancy is treated with radiotherapy if it is diagnosed at an early stage, however, at later stages treatment involves surgery often in conjunction with radiotherapy (Brook, 2013). The majority of head and neck cancers are diagnosed at an advanced stage; for example 62% of new diagnoses in Northern Ireland are reported to be at Stage III or Stage IV (Stage IV alone accounts for 45%) (Cancer Research UK, 2018). Late diagnosis means that many cases of laryngeal cancer have to undergo a partial or total laryngectomy. It has been estimated that there are currently 50,000–60,000 laryngectomees living in the United States (Brook, 2013).

Following a laryngectomy, the patient will no longer be able to form speech on their own. The gold standard for speech rehabilitation is tracheesophageal speech which requires the use of a small silicone valve known as a voice prosthesis (VP) (Bunting, 2004). Voice prostheses are susceptible to colonization by microbial biofilms (Busscher et al., 1997a). Such contamination may be enhanced by the environment of the esophagus, e.g., foods, liquids (including saliva), humidity and constant temperature (Holmes et al., 2014; Talpaert et al., 2015), and device failure has been attributed to such microbial growth as it can prevent valve closure (Leunisse et al., 2001; Oosterhof et al., 2005) and leakage of esophageal contents into the trachea. A loss of valve functionality leads to poor speech and requires replacement of the device (Ticac et al., 2010; Leonhard and Schneider-Stickler, 2015). Moreover, the microbial colonization may act as a reservoir of potential pathogens which, subsequent to aspiration, may lead to life-threatening infections such as pneumonia (Delank and Scheuermann, 2008). The mean VP lifespan is highly variable across different studies and has historically been reported as being between 120 and 200 days (Schafer et al., 2001; Sayed et al., 2012). However, a recent large-scale study which analyzed the lifespan of 3648 VPs reported an average of 86 days (Lewin et al., 2017). A number of strategies have been proposed to increase VP lifespan, such as the use of magnets to support the closing of the valve (Hilgers et al., 2003) and the incorporation of compounds, for example silver oxide, within the VP material to prevent microbial colonization (Hilgers et al., 2009). VPs containing magnets such as the Provox ActiValve have been shown to have longer *in situ* lifespans than other models (Kress et al., 2014; Lewin et al., 2017). However, overall, the levels of success with these approaches has been variable and importantly the average VP device lifespan has not significantly increased over the last 10 years (Lewin et al., 2017).

Co-colonization of VPs by bacterial and fungal species within poly-microbial biofilms is commonly reported. However, it is not clear to what extent yeast and bacterial biofilm colonization contributes to early VP failure, nor how effective their prevention may be in extending device lifespan. Medically, biofilms are of particular importance because it is thought that a significant percentage of human microbial infections include biofilm formation (Costerton et al., 1999; Donlan, 2001a,b). Furthermore, cells within biofilms can be up to 1000x more resistant to antimicrobial treatment than their planktonic counterparts (Chandra et al., 2001; Donlan and Costerton, 2002). The exact microbial composition on any particular VPs is influenced by several factors including patient diet, lifestyle and the voice prosthesis management regime to which they subscribe (Talpaert et al., 2015).

*Streptococcal* species such as *Streptococcus mitis* and *Streptococcus sobrinus*, *Staphylococcal* species such as *Staphylococcus aureus* (Neu et al., 1994), and *Pseudomonas aeruginosa* (Sayed et al., 2012) are among the major bacterial microorganisms frequently isolated from VPs biofilms. *Candida albicans* is reported as the most prevalent fungal colonizer of a VP surface (Bauters et al., 2002; Buijssen et al., 2012), it is a commensal yeast that is found on the mucosal surfaces of the oral cavity, gastrointestinal tract and genitourinary tract of most healthy individuals (Berman and Sudbery, 2002; Ganguly and Mitchell, 2011). *C. albicans* is a dimorphic fungus and biofilm formation of this organism involves a switch from growing in the typical budding yeast form to a filamentous hyphal form.

Here we characterize the microflora found on early failing voice prosthesis from 48 patients within Kent (South East United Kingdom) over a 5-year period (2011–2016). We observed that multi-species biofilms form in most cases, with *C. albicans* and *S. aureus* being the most prevalent. We demonstrate that the high CO_2_ environment experienced in the airway promotes *C. albicans* biofilm formation on VPs, providing an explanation for its prevalence on these devices. To determine whether yeast colonization acts as a driver for early VP failure we devised an evidence based strategy to manage contamination using a newly developed high-throughput assay that mimics *in situ* conditions in combination with drug sensitivity data. We report results from a 20 patient study that applies our principles of precision antimicrobial assessment that verifies antifungal treatment as a highly effective approach to VP lifespan extension. The study documented voice prosthesis lifespan pre- and post-management pathway implementation and statistical analyses confirmed a significant increase in VP lifespan within the patient cohort. Our guidelines represent a significant advance in both our understanding of the underlying cause of VP failure and a new effective method to increase VP lifespan by an average of 2.7-fold.

## Materials and Methods

### Patient Cohort and Voice Prostheses

Biofilms from a total of 159 early failing VPs from 48 patients (41 males and 7 females; mean age = 69.5 years, range 35–90 years) were analyzed for microorganism composition over a 5-year period (2011–2016). All prostheses were removed from patients attending one of the three main acute hospital sites within East Kent, United Kingdom: William Harvey Hospital, Ashford; Canterbury Hospital, Canterbury; Queen Elizabeth the Queen Mother Hospital, Margate. 38 of these patients had multiple voice prosthesis failures and were followed as part of a *Candida* management guideline impact study, which documented the voice prosthesis lifespans before and after implementation of a set of treatment guidelines designed to limit fungal growth. Treatment guidelines for the treatment of VPs with anti-fungal drugs were approved by the East Kent Hospital University Foundation Trust (EKHUFT) Voice Prosthesis Infection Management Multi-disciplinary team (MDT), EKHUFT Antimicrobial Stewardship Group, EKHUFT Drugs and Therapeutics Committee, EKHUFT ENT Audit Group, EKHUFT Adult Speech and Language Therapy Service, East Kent Prescribing Group. This study was approved by the EKHUFT Research and Innovation Department (2018/GAP/20) in accordance with the Department of Health’s Research Governance Framework for Health and Social Care and EKHUFT Research and Innovation policy. All patient data was anonymised prior to use in this study.

Please contact the corresponding authors for information if you wish to start using the *Candida* Management clinical guidelines for voice prosthesis management used in this study.

### Microorganism Isolation and Identification

To determine which microorganisms were present, a failed voice prosthesis was removed from the patient and sealed in a sterile bag. Upon receipt at the microbiology laboratory, the voice prosthesis was added to 2 ml saline solution along with glass beads and vortexed at 2500 rpm for 30 s. The resulting suspension was plated on chromogenic agar to facilitate identification of bacterial species. 50 μl of the suspension was also plated on Sabouraud Dextrose Agar (SDA) plates (Oxoid, CM0041) containing 100 μg/ml chloramphenicol (Oxoid, SR0078) to promote the growth of fungal species while inhibiting bacterial growth. Chromogenic agar and SDA plates were incubated for 48 h at 37°C. Bacterial colonies were then picked from the chromogenic plates and fungal colonies were picked from the SDA plates, species were identified using matrix assisted laser desorption ionization time-of-flight mass spectrometry (MALDI-ToF).

### MALDI-ToF Mass Spectrometry Identification of Microorganisms

MALDI-ToF mass spectrometry was used to identify species isolated from failed VPs within this study. Fungal and bacterial colonies were picked and applied to a steel MALDI target plate before being overlaid with an α-cyano-4-hydroxycinnamic acid (4-HCCA) matrix (Sigma-Aldrich, 70990) (Gobom et al., 2001). MALDI-ToF mass spectrometry was performed using a Bruker MALDI Biotyper (Bruker) as per the manufacturer’s instructions. The resulting mass spectra were compared to a reference database for identification using the MBT Compass and MBT Explorer Software along with the MBT Compass Library which comprises approximately 2750 species from 471 microorganism genera.

### Antifungal Sensitivity Testing

Antifungal sensitivity testing was performed using the Fungitest^TM^ commercial testing kit (BioRad, 60780) as per the manufacturer’s instructions. Isolates were assigned sensitive, intermediate, or resistant based upon the European Committee on Antimicrobial Sensitivity Testing (EUCAST) breakpoint recommendations for each antifungal and/or species (European Committee on Antimicrobial Susceptibility Testing, 2018).

### Antibacterial Sensitivity Testing

Antibacterial sensitivity testing was carried out to determine the minimum inhibitory concentrations (MICs) of clinically commonly used antibiotics. Isolates were assigned sensitive, intermediate, or resistant based upon the EUCAST breakpoint recommendations for each antibiotic and/or species (European Committee on Antimicrobial Susceptibility Testing, 2019).

### Scanning Electron Microscopy (SEM) of Voices Prosthesis Surfaces

Segments were taken from the valve and flange of a Provox Vega voice prosthesis and mounted onto 12.5 mm aluminum SEM specimen stubs (Agar Scientific, AGG301) with superglue. The surfaces were imaged at ambient temperature with a Hitachi S-3400N scanning electron microscope, using the variable pressure scanning electron microscopy (VP-SEM) mode with a chamber pressure of 30 Pa and accelerating voltage of 10 kV. The backscattered electron (BSE) detector in conjunction with the energy dispersive X-ray spectroscopy (EDS) detector was used throughout with a working distance of 10 mm. The acquisition software was Oxford Instruments INCA and images were exported directly from this.

### Atomic Force Microscopy (AFM) of Voice Prosthesis Surfaces

Segments were taken from the valve and flange of a Provox Vega voice prosthesis and mounted onto 15 mm AFM specimen disks (Agar Scientific, F7003) with superglue. These surfaces were imaged at ambient temperature using a Bruker Multimode 8 scanning probe microscope with a Nanoscope V controller, using the ScanAsyst peak-force tapping mode with a 50 μm × 50 μm scan area and a 700 nm scan height. SCANASYST-AIR (Bruker) silicon nitride cantilevers (tip height of 2.5–8.0 μm, nominal tip radius of 2 nm) with a nominal spring constant of 0.4 N/m and a resonance frequency of 70 kHz were used throughout. The image acquisition software was Nanoscope 8.15 R3sr5 and the processing software was Nanoscope Analysis.

### *Candida* Strains and Growth Media

*Candida* strains (Supplementary Table S3) were routinely grown at 30°C in yeast peptone dextrose (YPD) media (2% peptone (BD Bacto), 2% D-glucose (Fisher Scientific), 1% yeast extract (BD Bacto). For the biofilm growth assays, *Candida* biofilms were grown at 37°C in RPMI-1640 media (Sigma-Aldrich, R8755) supplemented with 80 μg/ml uridine (Sigma-Aldrich, U3750). *Candida* strains were maintained in YPD + 20% glycerol at −80°C for long-term storage and revived at 30°C on YPD + 2% Technical Agar (Oxoid) plates.

### *In vitro* Biofilm Growth Assays

To investigate fungal biofilm formation and its treatment on VPs a high-throughput assay was developed. *C. albicans* biofilms were grown on a PDMS silicone elastomer (Provincial Rubber, S1). The silicone was cut into 1 cm^2^ squares and placed in clips in a modified 24-well plate lid (Academic Centre for Dentistry Amsterdam, AAA-model) so they could be suspended in media within a sterile 24-well plate (Greiner Bio-one, CELLSTAR, 662160). Silicone squares were incubated in 1 ml 50% donor bovine serum (DBS) (Gibco, 16030074) for 30 min at 30°C, then washed twice with 1 ml Phosphate-Buffered Saline (PBS – 137 mM NaCl, 2.7 mM KCl, 10 mM Na_2_HPO_4_, 1.8 mM KH_2_PO_4_, pH 7.4) to remove excess DBS. *Candida* strains were inoculated into a test tube containing 5 ml YPD media and placed in a 30°C orbital shaking incubator with constant shaking at 180 rpm for 18 h. OD_600_ measurements were taken and a volume of overnight culture corresponding to OD_600_ = 1.0 (3 × 10^7^ CFU/ml) was removed. These cells were pelleted by centrifugation at 4000 rpm for 5 min at which point the supernatant was discarded. The resultant pellet was re-suspended in 5 ml PBS to wash and centrifuged again at 4000 rpm for 5 min. The PBS supernatant was discarded and the pellet re-suspended in fresh PBS (at an OD_600_ of 1.0). The OD_600_ 1.0 standard cell suspension was added to wells (1 ml per well) in a pre-sterilized 24-well plate and the lid with the silicone squares attached was placed on top so the silicone squares protruded into the cell suspension. These plates were then incubated at 37°C (in either 0.03% CO_2_ or 5% CO_2_) without shaking for 90 min to allow cell attachment to the silicone. After the attachment phase, the silicone squares were washed twice with 1 ml PBS to remove any unattached cells and transferred to 1 ml RPMI-1640 media (Sigma-Aldrich, R8755). They were then incubated at 37°C (in either 0.03% CO_2_ or 5% CO_2_) without shaking for up to 48 h to allow biofilm maturation. Growth of biofilms on the surface of a sterile VP was conducted using the same protocol.

### Biofilm Quantification via XTT Assay

Biofilm growth was quantified using an XTT assay (Kuhn et al., 2002). Biofilms were washed twice with 1 ml PBS to remove any planktonic cells before proceeding to quantification. After washing, the biofilms were transferred to a new pre-sterilized 24-well plate (Greiner Bio-one, CELLSTAR, 662160) containing 30 μg/ml XTT labeling reagent (Roche, 11465015001) and incubated at 37°C for 4 h. After incubation, the biofilms were removed from the 24-well plate and the absorbance of the remaining XTT labeling reagent was measured at 492 nm using a BMG LABTECH FLUOstar Omega plate reader machine.

### Antifungal Treatment of Biofilms

Biofilms were seeded on silicone elastomer sections as described above and grown in RPMI-1640 for 24 h at 37°C. The biofilms were then transferred to fresh RPMI-1640 media containing an antifungal, either; Fluconazole, Miconazole, or Nystatin at indicated concentrations. Fluconazole (Santa Cruz Biotechnology, sc-205698) was made as a 50 mg/ml stock solution in ethanol and diluted in RPMI-1640 final concentrations of 128 and 32 μg/ml. Miconazole (Santa Cruz Biotechnology, sc-205753) was made as a 50 mg/ml stock solution in DMSO and also diluted in RPMI-1640 to final concentrations of 32 and 128 μg/ml. Nystatin (Santa Cruz Biotechnology, sc-212431) was made as a 5 mg/ml stock solution in DMSO and diluted in RPMI-1640 to final concentrations of 2 and 8 μg/ml. Drug vehicle controls (0.25% ethanol for Fluconazole, 0.25% DMSO for Miconazole, and 0.15% DMSO for Nystatin) were used to ensure the solvents were not affecting biofilm growth. The biofilms matured in the RPMI-1640 media containing the select antifungal for a further 24 h at 37°C in both 0.03 and 5% CO_2_ before proceeding to quantification via the XTT assay. Experiments were performed in biological and technical triplicate.

### Mixed Species Biofilm Competition Assays

Biofilms were set up as described previously, except that for mixed biofilms *C. albicans* and *C. parapsilosis* clinical isolate overnight cultures were each counted and adjusted to 1.5 × 10^7^ CFU/ml to give a 3 × 10^7^ CFU/ml overall inoculum (equivalent to OD_600_ of 1.0) for the 90 min attachment phase. After attachment, the silicone squares were washed twice with 1 ml PBS to remove any unattached cells and transferred to 1 ml RPMI-1640 media (Sigma-Aldrich, R8755). They were then incubated at 37°C in both 0.03% CO_2_ or 5% CO_2_ without shaking for 48 h to allow biofilm maturation before proceeding to quantification via the XTT assay. Experiment was performed in biological and technical triplicate.

### Biofilm Composition Analysis via Chromogenic Agar

Silicone squares with biofilms on them were dropped into 2 ml PBS and vortexed for 10 s at 2500 rpm to release the biofilm cells from the surface. The resulting biofilm cell suspension was plated on Candida Ident Agar (Fluka Analytical, 94382) in triplicate (200 μl per plate). The chromogenic agar plates were incubated at 30°C for 48 h, at which point photographs of the plates were taken. *C. albicans* colonies appeared green and *C. parapsilosis* colonies stayed white on the Candida Ident Agar plates.

### Analysis of Voice Prosthesis Lifespans

Voice prosthesis lifespans at the Speech and Language Therapy Centre at the Kent and Canterbury Hospital were investigated over the course of 8 years (2010–2018). In total, 38 patients had their voice prosthesis lifespan documented. However, 18 patients were removed from further analysis due to a lack of adherence to the guidelines, moving out of the hospital catchment area resulting in incomplete data entries, or simply not enough VPs failures/changes in the time period. The voice prosthesis lifespans of the remaining 20 patients, before and after the implementation of our treatment pathway, were analyzed using the Online Application for Survival Analysis 2 (OASIS 2) platform (Kim et al., 2016). In total, 143 VPs before and 176 after implementation of the pathway were included in this study. Kaplan–Meier survival curves were plotted and log-rank tests performed to statistically compare device lifespans before and after the treatment pathway. Wilcoxon Signed-Rank tests were also carried out to determine if the pathway effect was significant when taking into account the inherent differences in device lifespan from patient to patient. For all analyses, the significance level (α) was 0.05.

## Results

### Analysis of Microorganisms Found on Early Failing Voice Prostheses

**FIGURE 1:**
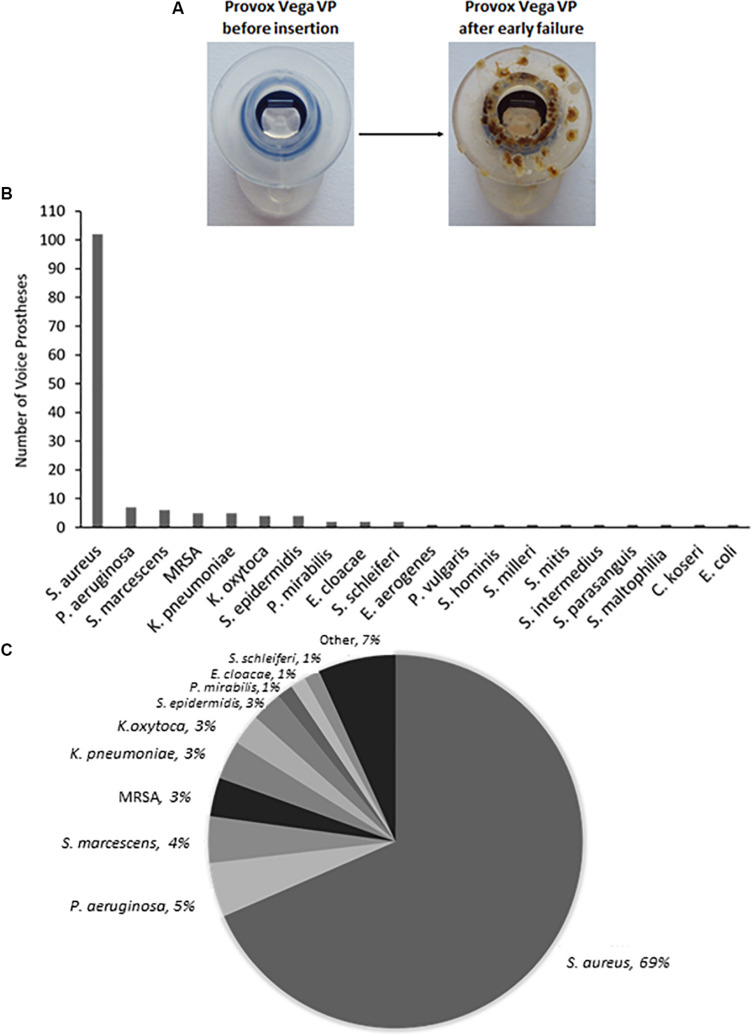
The range and distribution of bacterial species found on voice prostheses within this study. **(A)** An example of a failed voice prosthesis removed from a patient. Note the ring of colonization on the prosthesis hood around the valve mechanism. **(B)** Bar graph representation of absolute numbers of times each bacterial species was isolated from 159 early failing VPs. **(C)** Pie chart representation of percentage of total bacteria isolated from 159 early failing VPs.

*Staphylococcus aureus w*as the most frequently isolated bacterial species (Figure 1B), being found on multiple early failing VP (Figures 1B,C). This finding is in line with previous studies that also identified *S. aureus* as the prevalent bacterial organism found within VP biofilms (Neu et al., 1994; van Weissenbruch et al., 1997). However, *S. aureus* was one of several *Staphylococcal* species identified, the others being identified at lower frequencies included *Staphylococcus epidermidis* and *Staphylococcus schleiferi* (Figures 1B,C). We also identified the *S. aureus* strain methicillin-resistant *S. aureus* (MRSA) on 5 VPs and counted these separately from the rest of the *S. aureus* isolates (Figures 1B,C). *P. aeruginosa* was the second most frequent bacterial species found on early failing VPs (Figures 1B,C). Previous studies have also identified *P. aeruginosa* as a prevalent bacterial species found within VP biofilms (Sayed et al., 2012). Several *Streptococcus* species were also identified, but at a low frequency, including *Streptococcus milleri*, *Streptococcus mitis*, and *Streptococcus intermedius* (Supplementary Table S1).

**FIGURE 2:**
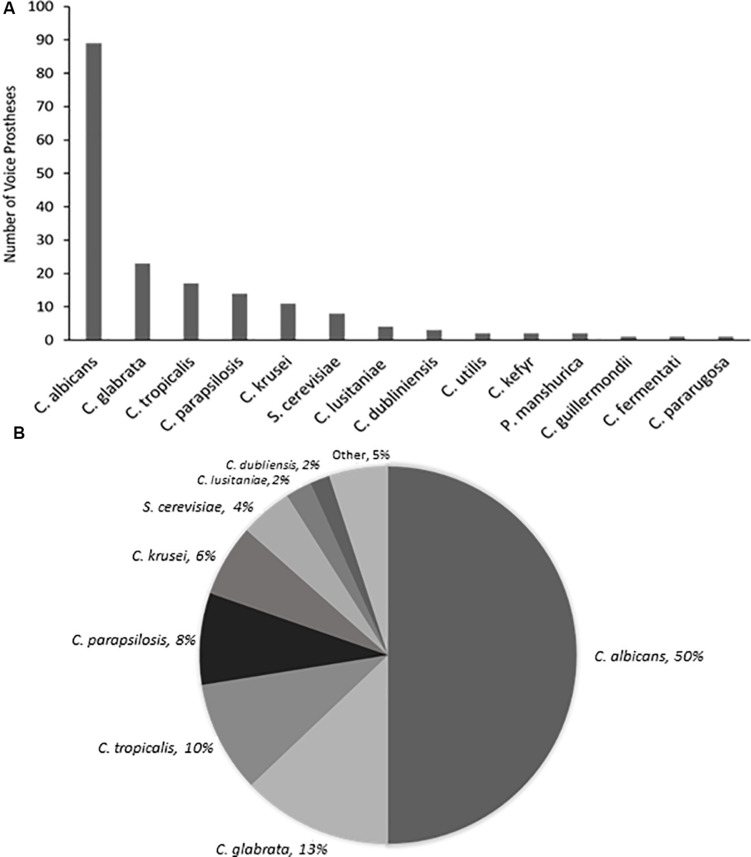
The range and distribution of fungal species found on early failing VP. **(A)** Bar graph representation of absolute numbers of times each yeast species was isolated from 159 early failing VPs. **(B)** Pie chart representation of percentage of total yeast isolated from 159 early failing VPs.

Yeast species were isolated from the vast majority of the 159 early failing VPs; 107 (67.3%) were colonized by a single yeast species, 37 (23.3%) were colonized by multiple yeast species, and just 15 (9.4%) showed no yeast species presence. In total, 178 yeast isolates were identified and 168 (94.4%) of these were *Candida* species, the remaining 10 fungal isolates were made up of *Saccharomyces cerevisiae* (8) and *Pichia manshurica* (2). *S. cerevisiae* and *P. manshurica* isolates were normally found alongside *Candida* species (62.5 and 100% respectively). The most frequently isolated yeast species was *C. albicans* (Figures 2A,B). This is in line with previous studies which identified *C. albicans* as the yeast species most commonly found on VPs (Everaert et al., 1997; Bauters et al., 2002; Buijssen et al., 2012). The non-*albicans Candida* isolates constituted 44.4% of all fungal isolates, with *Candida glabrata* being the most common of these (Supplementary Table S2).

The same bacterial and fungal species were frequently isolated from sequential VPs removed from the same patient implicating a common pattern of biofilm establishment. In total, 38 of the 48 patients had more than one voice prosthesis failure during the course of the study. 30 of these (78.9%) had the same bacterial species present on at least 2 VPs while 29 (76.3%) had the same fungal species on at least 2 VPs. 25 of the 29 patients which had reoccurring fungal species also had the same bacterial species on at least 2 VPs. The most commonly reoccurring fungal species was *C. albicans*, being found on multiple VPs within 18 patients.

It is worth noting that fungal and bacterial species were often found together on early failing VPs, 134 (84.3%) had at least one species of each present. The two most numerous species in this study, *S. aureus* and *C. albicans*, were the most commonly co-isolated species, being found together on 60 (37.7%) VPs. It was very rare for only fungal species to be present; only 10 (6.3%) VPs harbored solely fungal species and intriguingly all of these were either the first or second VP failure of their respective patients. The significance of this, if any, is yet to be determined. The two most commonly co-isolated fungal species were *C. albicans* and *C. glabrata* which were found together on 17 (10.7%) VPs.

It is of importance to highlight that the most prevalent voice prosthesis colonizing organisms within our patient cohort correlate with previous findings. This suggests that an effective treatment plan designed to tackle biofilm formation on VP in our cohort may have wide-reaching applicability.

### Factors Promoting Microbial Colonization of Voice Prostheses

**FIGURE 3:**
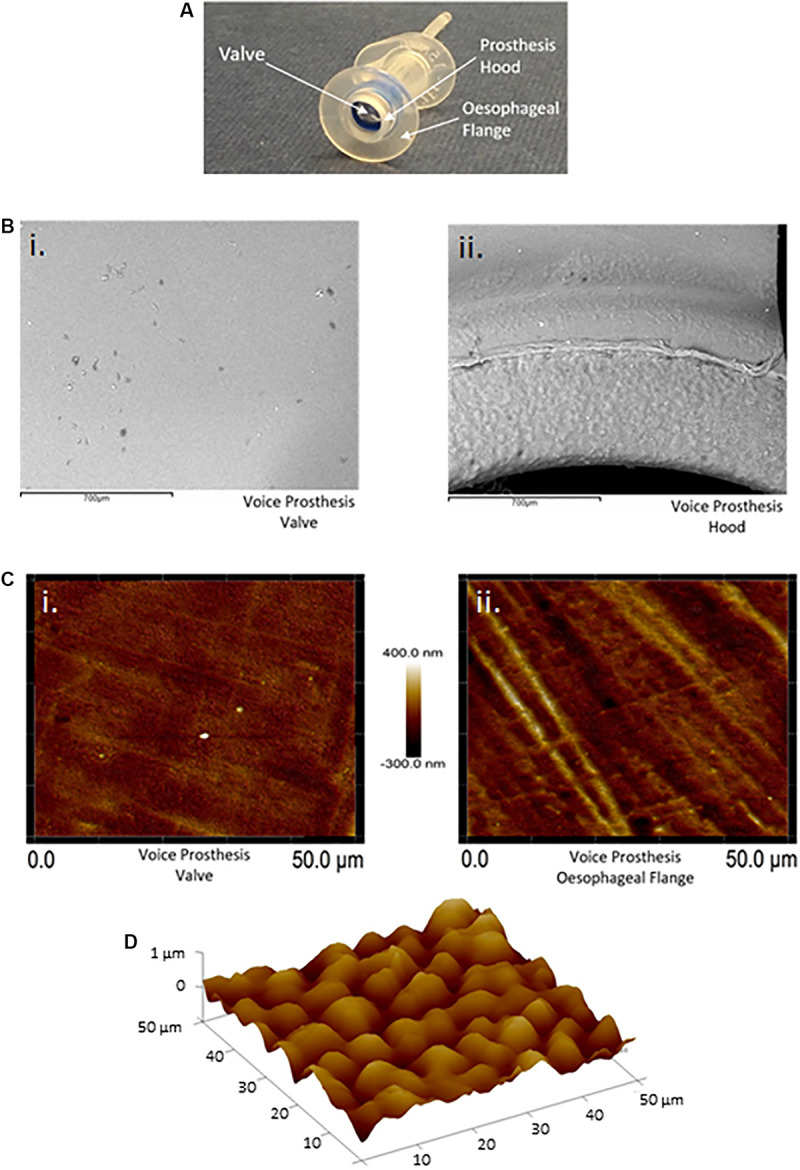
Atomic force and scanning electron microscopy surface topography of the valve and esophageal flange of the Provox Vega voice prosthesis. **(A)** The Provox Vega voice prosthesis. **(B)** SEM images of the valve and inner-side of the prosthesis hood were taken at x120 magnification. The scale bars represent 700 μm. **(C)** AFM images of the valve and esophageal flange were taken of 50 μm × 50 μm surface areas with a scan height of 700 nm. **(D)** AFM 3D plot of a 50 μm × 50 μm area of the esophageal flange. Z-axis is 0–1 μm. Several images were taken and representative examples are presented.

Our routine examination of early failing VPs often suggested concentration of microbial growth on certain regions of the device surface (Figure 1A). We investigated surface topography using atomic force and scanning electron microscopy as roughness has been shown to be an important driver of colonization (Radford et al., 1998; Nevzatoğlu et al., 2007). The flange (Figure 3A) of the device exhibited a significantly rougher topography than the valve which appeared smooth using these high resolution techniques (Figures 3B,C). The roughness of the flange was particularly evident in the AFM 3D plot of its surface (Figure 3D). In line with previous studies, this may suggest that the flange, specifically the valve-flange interface, provides a more likely site for initial attachment (Leunisse et al., 2001). This proposition is also consistent with our observations that failed VPs often exhibit heavy colonization on the flange, particularly at the rougher inner edge where the flange interfaces with the valve (Figures 1A, 3Bii).

**FIGURE 4:**
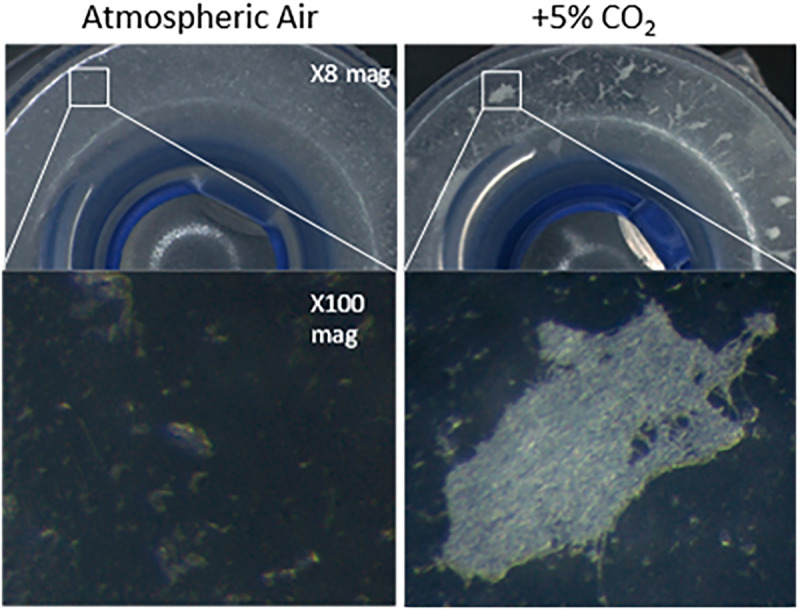
*Candida albicans* biofilm forming on the flange of a voice prosthesis *in vitro* in both atmospheric air and elevated CO_2_ conditions. *C. albicans* biofilm formation was assessed on a Provox Vega VP at 37°C under atmospheric air and elevated CO_2_ conditions that mimic exhaled breath. Images were collected after 24 h at x8 and x100 magnification. The experiment was repeated three times and representative images are presented.

Due to the high CO_2_ levels (∼5%) experienced in the airway as a result of exhaled breath (Tsoukias et al., 1998), we contemplated whether CO_2_ could be exerting an effect on *C. albicans* biofilm formation in the voice prosthesis scenario. Indeed, when seeded onto a voice prosthetic surface, *C. albicans* has increased biofilm growth in 5% CO_2_ after 24 h than in atmospheric air (Figure 4). Constant exposure of the valve mechanism and parts of the esophageal flange to exhaled air may therefore contribute to the prevalence of *C. albicans* colonization upon VPs.

### *C. parapsilosis* Does Not Have a Competitive Advantage Over *C. albicans* in Biofilm Establishment

**FIGURE 5:**
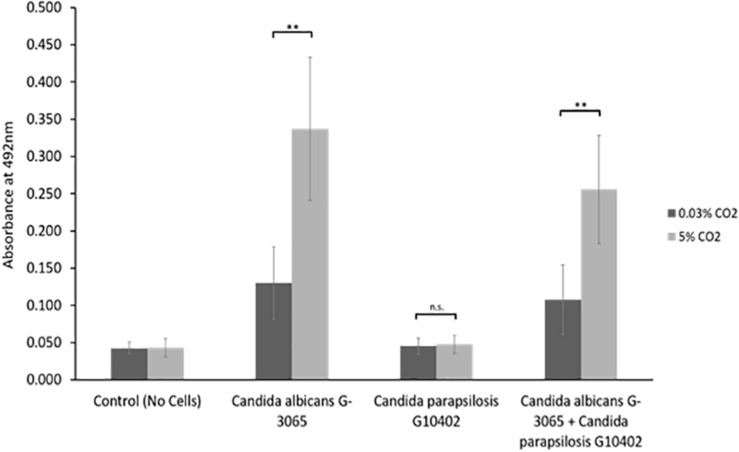
*In vitro* biofilm assay using *C. albicans* and *C. parapsilosis* early failing VP clinical isolates. Biofilms were seeded and grown for 48 h to mature, the resulting biofilms were quantified using the XTT assay which produces a red product that can be measured spectrophotometrically at 492 nm (higher cell number = higher absorbance). The *C. albicans* G-3065 clinical isolate makes bigger biofilms than the *C. parapsilosis* G10402 clinical isolate, and this biofilm formation is influenced by CO_2_ concentration. The graph represents three independent experiments each containing triplicates, error bars denote Standard Deviation. Paired two-tail *t*-tests were carried out: *p < 0.05, **p < 0.01, ***p < 0.001, n.s. = not significant.

We observed that *Candida parapsilosis* was rarely co-isolated with another *Candida* species. Of the 14 VPs which harbored *C. parapsilosis*, only 1 also had another *Candida* species present. This is a stark contrast to other *Candida* species, such as *C. glabrata* which was found on 23 VPs, 19 of which also had additional *Candida* species. This led us to investigate whether *C. parapsilosis* exhibited a competitive advantage over *C. albicans* with regards to biofilm growth. A *C. parapsilosis* clinical isolate found as the sole yeast species on a failed VP was selected for this investigation. Biofilms were seeded using equal cell numbers of the *C. albicans* and *C. parapsilosis* clinical isolates either alone or in combination and incubated for 48 h to mature. Overall biofilm growth was analyzed by measuring the metabolic activity of the biofilms using the XTT colorimetric assay (Kuhn et al., 2002) (Figure 5). The proportions of *C. albicans* and *C. parapsilosis* cells were then analyzed by removing biofilms from the silicone surface and plating the resulting cell suspension on chromogenic agar (Supplementary Figure S1). Strikingly, while the *C. albicans* G-3065 clinical isolate was able to form a robust biofilm on the silicone surface, the *C. parapsilosis* G10402 clinical isolate was not (Figure 5). Moreover, when these two clinical isolates were seeded together, the resultant biofilm was composed primarily of *C. albicans* cells (Supplementary Figure S1). In addition, while *C. albicans* biofilms were significantly increased when grown in the presence of increased CO_2_ this was not observed for *C. parapsilosis*. These data suggest that the identification of *C. parapsilosis* in isolation on failed VPs is unlikely to arise from a competitive advantage over *C. albicans*.

### Antimicrobial Sensitivity of Clinical Isolates

The sensitivities of the five most common bacterial species found on early failing VP to an example antibiotic from the five major antibiotic classes (Coates et al., 2011) are reported (Table 1). Only 10 (8.5%) of the most common bacterial isolates tested for sensitivity against the β-lactam antibiotic amoxicillin were judged to be sensitive, and these were all *S. aureus* isolates. All of the *P. aeruginosa*, *S. marcescens*, MRSA, and *K. pneumoniae* isolates tested for amoxicillin sensitivity were resistant. 76 (74.5%) were deemed to be sensitive to the macrolide erythromycin, with a significant number of *S. aureus* isolates being found to be resistant. The majority of the common bacterial isolates were sensitive to the fluoroquinolone Ciprofloxacin with 111 (93.3%) being judged as sensitive, however, all of the MRSA isolates tested were resistant to Ciprofloxacin. Likewise, a high number of isolates were sensitive to Tetracycline (104–87.4%), but this antibiotic did not exhibit good activity against the *P. aeruginosa* and *S. marcescens* isolates with 5 (83.3%) of both species being resistant. Finally, 115 (96.6%) of the common bacterial isolates tested were sensitive to the aminoglycoside Gentamicin, this was in fact the most effective antibiotic tested in this study (Table 1).

**TABLE 1:** Antibiotic sensitivity of the five most common bacterial species isolated from early failing VPs.

**Species**	**Total number of isolates tested**	**Number of isolates in each category (%)**		
		**Sensitive**	**Intermediate**	**Resistant**
**Amoxicillin**				
*Staphylococcus aureus*	96	10 (10.4)	0	76 (79.2)
*Pseudomonas aeruginosa*	6	0	0	6 (100)
*Serratia marcescens*	6	0	0	6 (100)
MRSA	5	0	0	5 (100)
*Klebsiella pneumoniae*	5	0	0	5 (100)
**Total**	**118**	**10 (8.5)**	**0**	**98 (83.1)**

**Erythromycin**				
*Staphylococcus aureus*	97	71 (73.2)	1 (1.0)	25 (25.8)
*Pseudomonas aeruginosa*	0	–	–	–
*Serratia marcescens*	0	–	–	–
MRSA	5	5 (100)	0	0
*Klebsiella pneumoniae*	0	–	–	–
**Total**	**102**	**76 (74.5)**	**1 (1.0)**	**25 (24.5)**

**Ciprofloxacin**				
*Staphylococcus aureus*	97	95 (97.9)	0	1 (1.0)
*Pseudomonas aeruginosa*	6	5 (83.3)	0	1 (16.7)
*Serratia marcescens*	6	6 (100)	0	0
MRSA	5	0	0	5 (100)
*Klebsiella pneumoniae*	5	5 (100)	0	0
**Total**	**119**	**111 (93.3)**	**0**	**7 (5.9)**

**Tetracycline**				
*Staphylococcus aureus*	97	95 (97.9)	0	2 (2.1)
*Pseudomonas aeruginosa*	6	0	0	5 (83.3)
*Serratia marcescens*	6	0	1 (16.7)	5 (83.3)
MRSA	5	5 (100)	0	0
*Klebsiella pneumoniae*	5	4 (80)	0	1 (20)
**Total**	**119**	**104 (87.4)**	**1 (0.8)**	**13 (10.9)**

**Gentamicin**				
*Staphylococcus aureus*	97	93 (95.9)	0	4 (4.1)
*Pseudomonas aeruginosa*	6	6 (100)	0	0
*Serratia marcescens*	6	6 (100)	0	0
MRSA	5	5 (100)	0	0
*Klebsiella pneumoniae*	5	5 (100)	0	0
**Total**	**119**	**115 (96.6)**	**0**	**4 (3.4)**

*The number of sensitive, intermediate, and resistant isolates based upon EUCAST breakpoint recommendations are given for the five most commonly isolated bacterial species.*

The sensitivities of the five most frequently isolated fungal species in this study to Fluconazole, Miconazole and Nystatin, three commonly used antifungals in the clinical setting, are reported (Table 2). Of the 168 *Candida* isolates, 155 were tested for Fluconazole sensitivity and 139 of these were found to be sensitive (89.7%). Moreover, 123 of the *Candida* isolates were tested for Miconazole sensitivity and 107 were sensitive (87.0%). Finally, 129 of the *Candida* isolates were tested for Nystatin sensitivity and 126 of these were found to be sensitive (97.7%). Nystatin therefore appeared to be the most effective antifungal with regards to inhibition of growth of *Candida* species obtained from early failing VPs in this patient cohort.

**TABLE 2:** Antifungal sensitivity of the five most common *Candida* species isolated from early failing VPs.

**Species**	**Total number of isolates tested**	**Number of isolates in each category *(%)***		
		**Sensitive**	**Intermediate**	**Resistant**
**Fluconazole**				
*Candida albicans*	83	81 (97.6)	2 (2.4)	0
*Candida glabrata*	23	21 (91.3)	2 (8.7)	0
*Candida tropicalis*	15	15 (100)	0	0
*Candida parapsilosis*	14	12 (85.8)	0	2 (14.3)
*Candida krusei*	9	1 (11.1)	2 (22.2)	6 (66.7)
**Total**	**144**	**130 (90.3)**	**6 (4.2)**	**8 (5.5)**

**Miconazole**				
*Candida albicans*	62	59 (95.1)	3 (4.9)	0
*Candida glabrata*	19	19 (100)	0	0
*Candida tropicalis*	13	7 (53.9)	6 (46.1)	0
*Candida parapsilosis*	9	4 (44.4)	4 (44.4)	1 (11.1)
*Candida krusei*	9	8 (88.9)	1 (11.1)	0
**Total**	**112**	**97 (86.6)**	**14 (12.5)**	**1 (0.9)**

**Nystatin**				
*Candida albicans*	65	64 (98.5)	0	1 (1.6)
*Candida glabrata*	20	18 (90.0)	1 (5.0)	1 (5.0)
*Candida tropicalis*	15	15 (100)	0	0
*Candida parapsilosis*	9	9 (100)	0	0
*Candida krusei*	9	9 (100)	0	0
**Total**	**118**	**115 (97.4)**	**1 (0.9)**	**2 (1.7)**

*The number of sensitive, intermediate, and resistant isolates based upon EUCAST breakpoint recommendations are given for the five most commonly isolated yeast species.*

### Antifungal Sensitivity of *C. albicans* Clinical Isolates Within Biofilms

**FIGURE 6:**
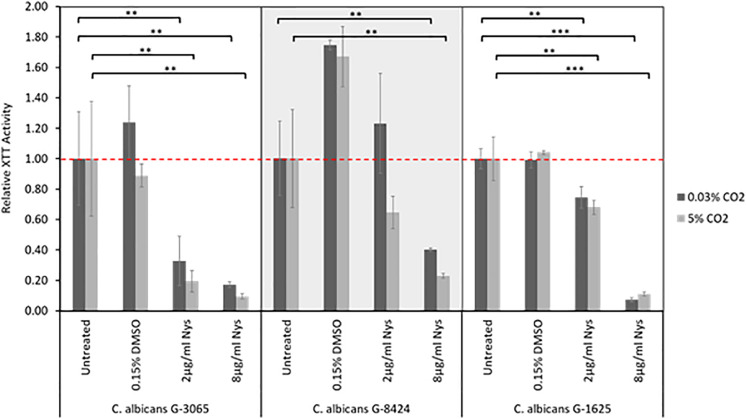
Nystatin sensitivities of *C. albicans* clinical isolate biofilms. Biofilms were seeded and grown for 24 h before addition of Nystatin, they were then grown for a further 24 h before quantification using the XTT assay. The relative XTT activity is presented with the 0.03% CO_2_ biofilms being normalized to the 0.03% CO_2_ untreated control and the 5% CO_2_ biofilms being normalized to the 5% CO_2_ untreated control. This prevents the general higher growth of 5% CO_2_ biofilms impacting the analysis. Graphs represent three independent experiments each containing triplicates, error bars denote Standard Deviation. Two-way ANOVAs followed by a Tukey test for multiple comparisons were carried out: *p < 0.05, **p < 0.01, ***p < 0.001. Stars directly above the bars indicate a significant difference to untreated in the same CO_2_ environment.

The antifungal drugs Nystatin, Miconazole, and Fluconazole are commonly used as agents to treat fungal colonization of the ear, nose, and throat. We therefore tested their efficacy against *C. albicans* biofilms grown from isolates recovered from early failing VPs. The clinical isolates displayed higher resistances to Fluconazole and Miconazole compared to Nystatin when growing as biofilms (Table 3). Moreover, this azole resistance was significantly increased in biofilms grown in high CO_2_ environments (Supplementary Figure S2). For instance, G-8424 biofilms grown in 0.03% CO_2_ had an average decrease in XTT activity of 59% relative to the untreated control upon treatment with 32 μg/ml Fluconazole (*p* < 0.001), whereas there was no significant decrease in relative XTT activity in the 5% CO_2_ G-8424 biofilms (Table 3 and Supplementary Figure S2). This was also true for the 128 μg/ml Miconazole treatments in all three isolates (Table 3 and Supplementary Figure S3). Furthermore, despite the fact that 0.03 and 5% CO_2_ G-1625 biofilms both displayed significant decreases in relative XTT activity upon Fluconazole treatment, the relative XTT activity was still significantly higher in the biofilms grown in 5% CO_2_ (Supplementary Figure S2). Nystatin sensitivities were generally independent of CO_2_ concentration (Figure 6). The G-3065 and G-8424 clinical isolate biofilms were more resistant to Fluconazole and Miconazole than the G-1625 isolate, however, all isolate biofilms exhibited good sensitivity to Nystatin (Table 3). Based on these data, we conclude that Nystatin is the most potent against *C. albicans* biofilms on silicone surfaces and also offers the greatest protection against the CO_2_ biofilm activation. However, Fluconazole and Miconazole are still both efficacious against some isolates. This approach, which extended current drug susceptibility testing practice to better represent *in vivo* biofilm growth conditions, was crucial in our decision to make use of Nystatin as the lead compound in the development of antifungal treatment guidelines.

**TABLE 3:** Antifungal sensitivity of biofilms of three *C. albicans* clinical isolates from early failing VPs.

**Antifungal**	***C. albicans* G-3065**		***C. albicans* G-8424**		***C. albicans* G-1625**	
	**0.03% CO_2_**	**5% CO_2_**	**0.03% CO_2_**	**5% CO_2_**	**0.03% CO_2_**	**5% CO_2_**
**Fluconazole**						
32 μg/ml	+	+	++	−	+++	++
128 μg/ml	+	+	+++	−	+++	++
**Miconazole**						
32 μg/ml	−	−	−	−	−	+
128 μg/ml	++	−	+++	−	+++	+
**Nystatin**						
2 μg/ml	+++	+++	−	+	+	+
8 μg/ml	+++	+++	++	+++	+++	+++

*Action of antifungals was measured via the XTT assay activity relative to untreated in both 0.03 and 5% CO_2_: − >80%, + 60–80%, ++ 40–60%, +++ <40%.*

### Development and Clinical Testing of Antifungal Treatment Guidelines (ATG) to Extend VP Lifespan

Although VP biofilms are likely to be formed from several species we hypothesized that an antifungal approach to reduce colonization may effective in extending device lifespan. The rationale behind this was based on three observations. First, yeast contamination contributes significant biomass which in turn is more likely to impair valve function. Second, the elevated physiological CO_2_ environment in which a VP sits promotes biofilm growth of the most commonly isolated yeast, *C. albicans*. Third, since other commonly isolated bacteria, such as *S. aureus* and *P. aeruginosa* have been reported to use *Candida* hyphae as a scaffold to attach to during biofilm formation (Harriott and Noverr, 2009), it may be the case that antifungal treatment also attenuates the bacterial colonization.

The antifungal drugs selected for treatment were chosen based upon the fungal sensitivity data with Nystatin being demonstrated to be the most effective as it had the lowest levels of resistance (Table 2). Previous studies have also identified that most non*-albicans Candida* species have higher azole MICs (Ribeiro et al., 2001; Singh et al., 2002), whereas all *Candida* species have low *in vitro* Nystatin MICs (Nenoff et al., 2016). Moreover, *C. albicans* sensitivity to Nystatin when in a biofilm appears to be unaffected by CO_2_ level (Figure 6). Our antifungal voice prosthesis management guidelines involve determining the identity and antimicrobial sensitivities of microorganisms present on a failed VP. Based on the antifungal sensitivities of the colonizing fungal species, a course of antifungals (most commonly Nystatin) is suggested and prescribed for topical application directly on the VP.

To test the effectiveness of our antifungal approach to managing VP data from 38 patients was assessed. VP lifespan data from the patient cohort before and after their placement on the ATG was assessed. Over the course of the study, 18 patients were removed from further analysis because of either; concerns over a lack of adherence to the ATG, moving out of the hospital catchment area resulting in incomplete data entries, or insufficient VP replacement data. This left 20 patients with complete VP lifespan data entries before and after guideline implementation on which to conduct statistical analyses.

Throughout the 8 year course of the study, the lifespan of 319 VPs (143 before and 176 after guidelines) across the 20 patient cohort were analyzed. These 319 VPs represented all the changes for the 20 patients when the removed VP was replaced by the same model of the same size. This ensured there were no external factors such as VP surface/surface area influencing the results. We also included all changes and not just those specifically attributed to the presence of *Candida* growth to remove bias.

**FIGURE 7:**
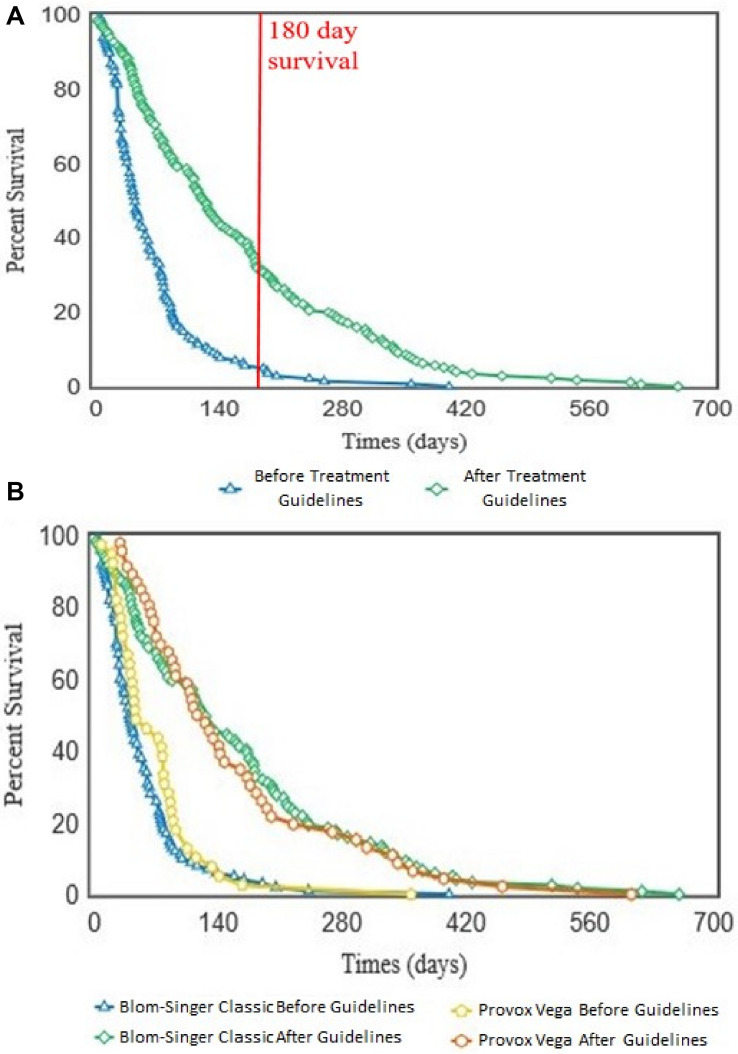
Kaplan–Meier survival curves showing the voice prosthesis *in situ* lifespans before and after implementation of our antifungal treatment pathway. **(A)** Device lifespans were documented before and after implementation of the ATG. Kaplan–Meier survival curves were plotted using the OASIS2 online platform and a log-rank test was used to compare the survival curves: *p* < 0.001. Red line is at 180 days lifespan which we targeted as the ideal lifespan to achieve. **(B)** After lifespan analysis across all devices was performed, the lifespans were categorized into groups depending on which VP model a patient was using. Kaplan–Meier survival curves were again plotted using the OASIS2 online platform and log-rank tests were used to compare them: Blom-Singer Classic, Before vs. After; *p* < 0.001. Provox Vega, Before vs. After; *p* < 0.001.

Overall, implementation of the ATG resulted in a significant (*p* < 0.001) increase in VP lifespan within our patient cohort (Figure 7A). Importantly, this lifespan increase was not dependent on VP manufacturer/model as both the Blom-Singer Classic and the Provox Vega (the two most common VPs in this study) exhibited similar increases in lifespan (Figure 7B). It is also important to highlight that the lifespans of these two models were not significantly different before or after the pathway implementation (Figure 7B).

**FIGURE 8:**
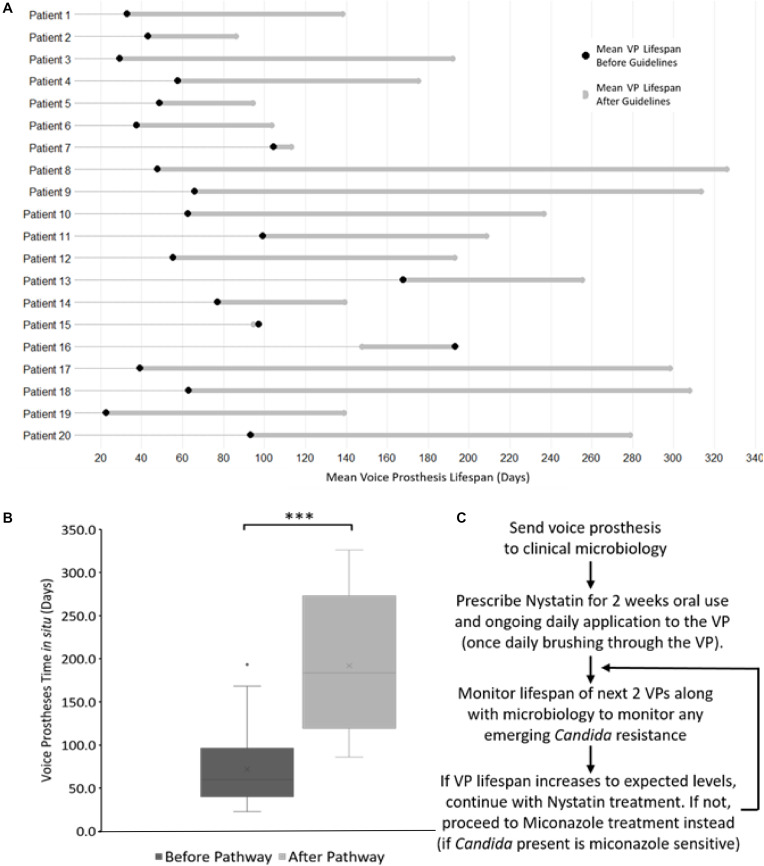
The antifungal treatment guidelines along with its effects on voice prosthesis lifespan within each patient. **(A)** Dumbbell chart representation of the mean VP lifespan difference after guideline implementation for each of the 20 patients. **(B)** Box plot representation of VP *in situ* lifespans before and after guideline implementation, Wilcoxon Signed-Rank test: ***p < 0.001. **(C)** Summary of the treatment pathway. As of January 2019, this management pathway has been distributed to 34 different speech and language therapy centers (32 in the United Kingdom, 2 internationally) upon request.

Although there was variation in average post-pathway VP lifespan increase between patients, the majority of the patients in our cohort enjoyed an overall increase in device lifespan (Figure 8A). Two patients did not exhibit an increase in VP lifespan, instead having a slight decrease of −2.4 and −45.5 days respectively (Figure 8A). However, these two patients had long-lived VPs on average (97.1 and 193.3 days respectively) in comparison with the rest of the cohort prior to the introduction of the new antifungal clinical guidelines. This was particularly evident for patient 16 whose VPs were already lasting longer than the post-pathway mean lifespan across all patients. Thus, the treatment guidelines may be of most benefit to patients who are experiencing early failing VP. The mean VPs lifespan before guideline implementation was 71.9 days and this increased to 192.0 days after implementation of the antifungal management pathway representing an average 2.7-fold increase in lifespan (Figure 8B).

We did not identify any cases of increased drug resistance to antifungal application within the study. However, our guidelines recommend a 6 months (180 days) limit before VP change. This length of time was chosen primarily to ensure that the VP remains structurally sound. Before ATG use only 8 VPs (5.6%) reached this target across all 20 patients, while post-ATG 63 VPs (35.8%) lasted at least 180 days (Figure 7A). It is worth noting that after the treatment guidelines were implemented, several VPs were being routinely changed because they had been *in situ* for >6 months and not because they had actually failed. The full set of guidelines can be obtained by contacting the authors and are briefly summarized in Figure 8C.

## Discussion

The colonization of VP by mixed biofilms has been reported as a potential cause for their early failure. However, a mechanistic understanding as to such biofilms may drive early failure and to what extend bacterial or fungal presence underlies failure is not well-understood. In this study we sought to investigate whether an antifungal treatment regime would provide an effective option for prevention early VP failure. Using a dedicated extraction protocol combined with high-powered microbiology identification we confirmed *Candida albicans* as the most prevalent fungal microorganism found on early failing VP. Our findings are in-line with previous studies where *C. albicans* was the most common fungal species isolated from VP (Buijssen et al., 2012). It is however likely that differences in the identification methods used, different patient demographics or lifestyles and different lifestyles or dietary habits will impact upon the microbial colonization of VP. For instance, Chaturvedi et al. (2014) found that *C. tropicalis* was the most prevalent fungal species colonizing VP within an Indian patient cohort. The high consumption of dairy products such as yogurt and buttermilk in India has been proposed to influence biofilm growth on VPs. For example, the presence of *Streptococcus thermophilus* and *Lactobacillus* in yogurt may reduce biofilm formation (Havenaar and Huis In’t Veld, 1992; Busscher et al., 1997b), while the high lactoferrin content (also found in saliva) in buttermilk has antibacterial and antifungal properties against organisms such as *C. albicans* and *Streptococcus mutans* (Soukka et al., 1991, 1992; Nikawa et al., 1993).

Our study found *S. aureus* as the most prevalent bacterial species on failed VPs, present on 64.2% of VPs, and was frequently found in combination with one or more *Candida* species. This may be expected as *S. aureus* is a member of the normal oral and perioral microbiota (McCormack et al., 2015). Moreover, it is the third most commonly isolated organism with *C. albicans* in poly-microbial infections (a significant proportion of which are nosocomial infections) (Klotz et al., 2007). Synergistic relationships between *S. aureus* and *C. albicans* have previously been documented. *S. aureus* is poor at forming biofilms on its own, however, it has been suggested that the hyphal *C. albicans* cells provide a scaffold on which the bacteria can attach within a poly-microbial biofilm (Harriott and Noverr, 2009). *S. aureus* has been proposed attach to *C. albicans* hyphae as they penetrate epithelial layers and this has been proposed as relevant for the invasion of human tissues during infection (Schlecht et al., 2015). Within a mixed biofilm, *S. aureus* cells have also been shown to become coated in *C. albicans* matrix material, enhancing its antibiotic resistance (Kong et al., 2016). Furthermore, *S. aureus* and *C. albicans* mixed biofilms can withstand higher shear stresses than pure *C. albicans* biofilms (Lin et al., 2013). Given the high frequency with which these two microorganisms are found together, along with the synergy they have been previously shown to exhibit, their inter-species relationship in with respect to biofilm formation on medical implant devices requires further investigation. However, it is quite plausible that by treating fungal contamination we may also decrease the burden of important bacterial pathogens, such as *S. aureus* on medical devices such as VPs. The potential impact of this relationship is highlighted by the fact co-infection results in increased mortality in mouse models than either microorganism alone (Carlson, 1982, 1983).

We have demonstrated that the lifespan of VP can be significantly increased by applying a highly effective personalized antimicrobial treatment regime focusing on *Candida* colonization, without directly treating the bacterial colonization. Initially we hypothesized that as VP failure occurs as a result of valve blockage then tackling the more significant biomass caused by yeast contamination then we may see better results than attempting to treat bacterial growth. However, our data also suggest that the rougher silicone surfaces of the VP and more significantly the high CO_2_ environment within exhaled breath can promote *Candida* biofilm establishment. Another important reason for tackling yeast contamination is that *C. albicans* has been shown to be able to degrading silicone rubber (Buijssen et al., 2012) which may contribute further to device failure. An antifungal approach may also be advantageous because bacteria such as *S. aureus* and *P. aeruginosa* have been reported to attach to *Candida* hyphae during biofilm formation (Harriott and Noverr, 2009), meaning antifungal treatment may also attenuate the bacterial colonization.

Prior to implementation of our VPs management pathway, the average *in situ* lifespan of VPs within our patient cohort was 71.9 days. This is similar to a recent large-scale study of voice prosthesis lifespans in the U.S. by Lewin et al. (2017) which found an average lifespan of 86 days. While being slightly less than a study by Kress et al. (2014) which found an average lifespan of 108 days. The average device lifespan within our patient cohort increased to 192 days after clinicians started following the management guidelines – a 270% increase. Importantly, the increase in lifespan was not affected by the make or model of VP within the study group, suggesting that this treatment regime may be applicable to a broad range of patients. The actual lifespan of VPs post-pathway may be higher than the 192 days reported here, as we recommended the clinicians at the Kent and Canterbury Hospital Speech and Language Therapy Centre change VPs prophylactically after 6 months (180 days) *in situ*. This ensures there is no biofilm formation around the TEP as well as no structural deterioration of the device due to mechanical stress and the constant application of antifungals. It should be noted that two of our patients did not experience an increase in VP lifespan while using the antifungal treatment guidelines; such a result may be expected within such a multi-factorial clinical scenario. In both cases the patients exhibited relatively long lived VPs before treatment began, and these serve to highlight the importance of patient monitoring and that fungal colonization may not be the cause of early VP failure in every case.

## Conclusion

*Candida albicans* was the most prevalent fungal species found on failed VPs within our patient group and we have demonstrated that the topical application of antifungals significantly extends voice prosthesis lifespan in the majority of patients. Our anti-fungal treatment guidelines have been fully ratified by the relevant EKHUFT NHS committees and have distributed widely throughout the United Kingdom. Given that several studies have identified similar microorganisms as predominant colonizers of VP it is possible that our ATG protocol may be widely applied and at time of submission it has been distributed to 34 speech and language therapy centers (32 in the United Kingdom and 2 internationally). Given the success of this approach it will be imperative to investigate the use of precision antifungal methodologies to tackle biofilm formation on a wider range of medical devices and tubing as a means to reducing infection, morbidity and mortality associated with their use.

## Data Availability Statement

All datasets generated for this study are included in the article/Supplementary Material.

## Ethics Statement

The studies involving human participants were reviewed and approved by East Kent Hospital University Foundation Trust (EKHUFT), EKHUFT Antimicrobial Stewardship Group, EKHUFT Drugs and Therapeutics Committee, EKHUFT ENT Audit Group, EKHUFT Adult Speech and Language Therapy Service, East Kent Prescribing Group. Written informed consent for participation was not required for this study in accordance with the national legislation and the institutional requirements.

## Author Contributions

This work was made possible through the collaborative efforts of the members of the *Candida* management multi-disciplinary team, all of whom played important roles and are placed as authors of this manuscript. DP performed data analysis, experimental procedures and was the primary author of this manuscript. CG, FM, AB, VM, and SS were responsible for conception of the study, experimental design, and editing of the manuscript. SS, LW, and CM were responsible for patient data collection. Microorganism identification from early failing VPs and initial antimicrobial sensitivity testing was carried out by MB.

## Conflict of Interest

The authors declare that the research was conducted in the absence of any commercial or financial relationships that could be construed as a potential conflict of interest.
