# Supplementary material for: Precision Antifungal Treatment Significantly Extends Voice Prosthesis Lifespan in Patients Following Total Laryngectomy
Source: Front Microbiol. 2020 May 20;11:975. doi: 10.3389/fmicb.2020.00975 (PMC7251058; doi:10.3389/fmicb.2020.00975)
Supplement: Supplementary file 1 [file Data_Sheet_1.PDF]

Figure S1

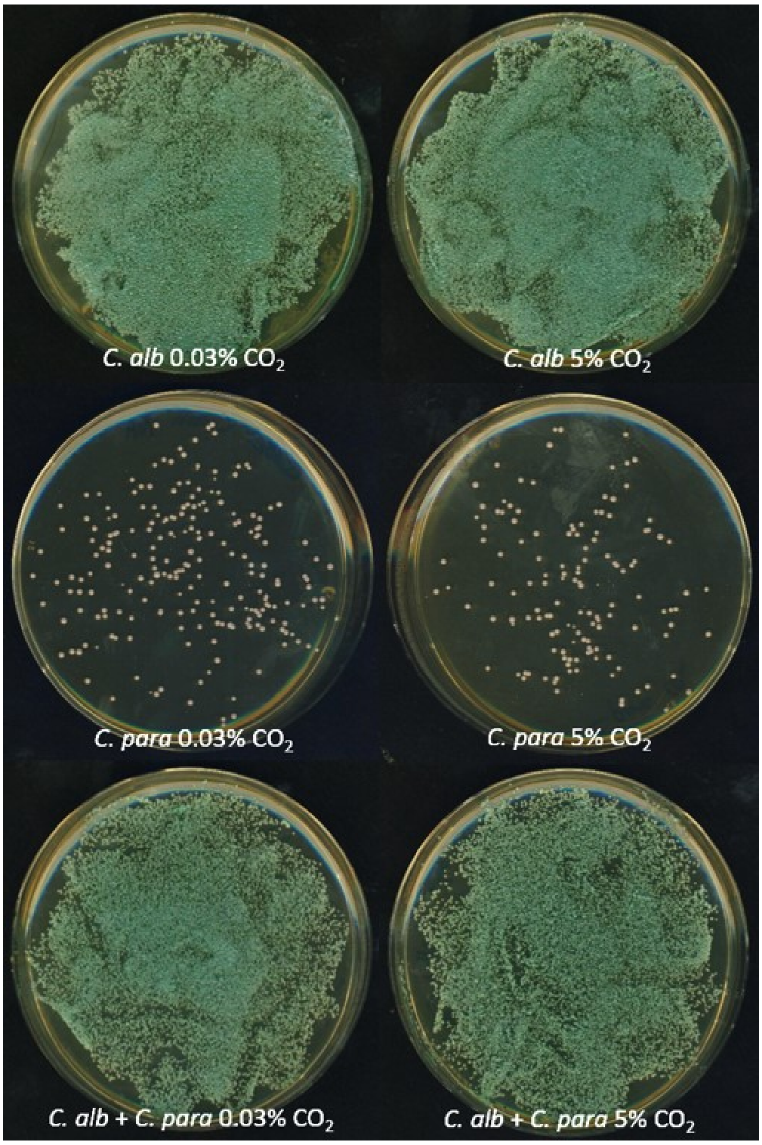

Figure S1: Chromogenic agar of *C. albicans* (G-3065) and *C. parapsilosis* (G10402) clinical isolate biofilms. Although they did not mature into a full biofilm, *C. parapsilosis* cells did attach to the silicone surface. The majority of the *C. albicans* and *C. parapsilosis* mixed biofilms are made up of *C. albicans* cells. Green colonies: *C. albicans*, white colonies: *C. parapsilosis*.

Figure S2

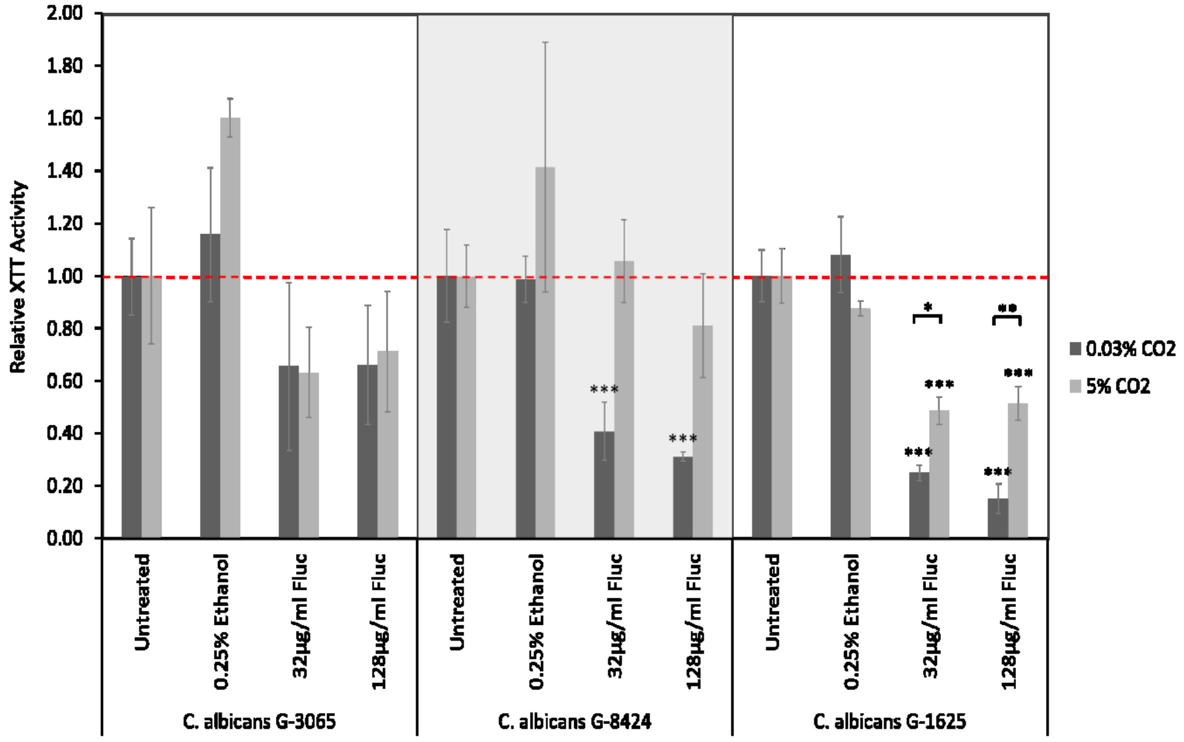

Figure S2: Fluconazole sensitivities of *C. albicans* clinical isolate biofilms. Biofilms were seeded and grown for 24 hours before addition of Fluconazole, they were then grown for a further 24 hours before quantification using the XTT assay. The relative XTT activity is presented with the 0.03% CO_2 biofilms being normalised to the 0.03% CO_2 untreated control and the 5% CO_2 biofilms being normalised to the 5% CO_2 untreated control. This prevents the general higher growth of 5% CO_2 biofilms impacting the analysis. Graphs represent three independent experiments each containing triplicates, error bars denote Standard Deviation. Two-way ANOVAs followed by a Tukey test for multiple comparisons were carried out: *$p<0.05$, **$p<0.01$, ***$p<0.001$. Stars directly above the bars indicate a significant difference to untreated in the same CO_2 environment.

Figure S3

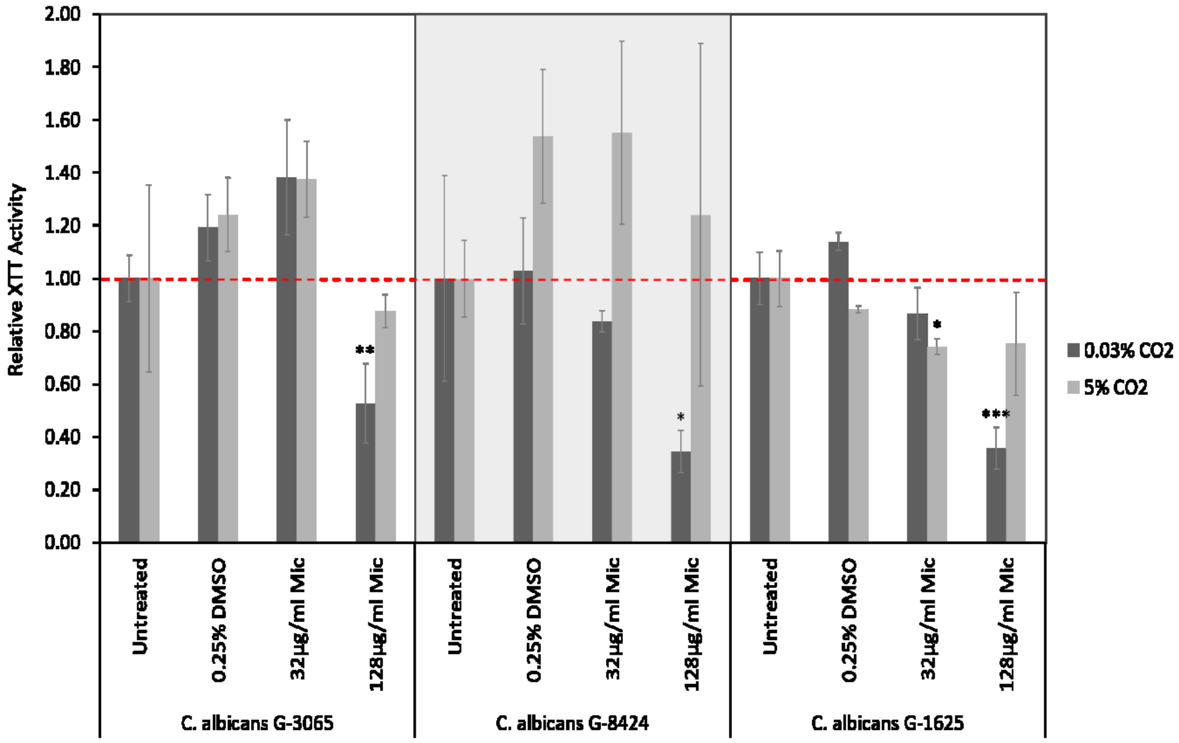

Figure S3: Miconazole sensitivities of *C. albicans* clinical isolate biofilms. Biofilms were seeded and grown for 24 hours before addition of Miconazole, they were then grown for a further 24 hours before quantification using the XTT assay. The relative XTT activity is presented with the 0.03% CO_2 biofilms being normalised to the 0.03% CO_2 untreated control and the 5% CO_2 biofilms being normalised to the 5% CO_2 untreated control. This prevents the general higher growth of 5% CO_2 biofilms impacting the analysis. Graphs represent three independent experiments each containing triplicates, error bars denote Standard Deviation. Two-way ANOVAs followed by a Tukey test for multiple comparisons were carried out: *$p<0.05$, **$p<0.01$, ***$p<0.001$. Stars directly above the bars indicate a significant difference to untreated in the same CO_2 environment.

Table S1

Species	Isolation Frequency from 159 Voice Prostheses	
	Number	Percentage
Staphylococcus aureus	102	64.2
Pseudomonas aeruginosa	7	4.4
Serratia marcescens	6	3.8
MRSA	5	3.1
Klebsiella pneumoniae	5	3.1
Klebsiella oxytoca	4	2.5
Staphylococcus epidermidis	4	2.5
Proteus mirabilis	2	1.3
Enterobacter cloacae	2	1.3
Staphylococcus schleiferi	2	1.3
Enterobacter aerogenes	1	0.6
Proteus vulgaris	1	0.6
Staphylococcus hominis	1	0.6
Streptococcus milleri	1	0.6
Streptococcus mitis	1	0.6
Streptococcus intermedius	1	0.6
Streptococcus parasanguis	1	0.6
Stenotrophomonas maltophilia	1	0.6
Citrobacter koseri	1	0.6
Escherichia coli	1	0.6

Table S1: Distribution of bacterial species on 159 voice prostheses. Number is the absolute number of voice prostheses upon which the particular species was found. Percentage is the percentage of voice prostheses upon which that species was found (not the percentage of total isolates).

Table S2

Species	Isolation Frequency from 159 Voice Prostheses	
	**Number**	**Percentage**
Candida albicans	89	56.0
Candida glabrata	23	14.5
Candida tropicalis	17	10.7
Candida parapsilosis	14	8.8
Candida krusei	11	6.9
Saccharomyces cerevisiae	8	5.0
Candida lusitaniae	4	2.5
Candida dubliniensis	3	1.9
Candida utilis	2	1.3
Candida kefyr	2	1.3
Pichia manshurica	2	1.3
Candida guillermondii	1	0.6
Candida fermentati	1	0.6
Candida pararugosa	1	0.6

Table S2: Distribution of fungal species on 159 voice prostheses. Number is the absolute number of voice prostheses upon which the particular species was found. Percentage is the percentage of voice prostheses upon which that species was found (not the percentage of total isolates).

Table S3

Strain	Species	Genotype/Parent	Source
**G-3065**	*C. albicans*	Clinical Isolate	Failed Voice Prosthesis
**G-8424**	*C. albicans*	Clinical Isolate	Failed Voice Prosthesis
**G-1625**	*C. albicans*	Clinical Isolate	Failed Voice Prosthesis
**G10402**	*C. parapsilosis*	Clinical Isolate	Failed Voice Prosthesis

Table S3: *Candida* strains used within this study.
